# A retrospective study on the association of gastrointestinal symptoms in children with low lactase activity and low activity of other disaccharidases

**DOI:** 10.1186/s12876-020-01443-4

**Published:** 2020-10-09

**Authors:** Paul Wasuwanich, Hassan Choudry, Thammasin Ingviya, Ann O. Scheimann, Karla J. AuYeung, Christine Karwowski, Susan Billet, Buford L. Nichols, Wikrom Karnsakul

**Affiliations:** 1 Division of Pediatric Gastroenterology, Hepatology, and Nutrition, Department of Pediatrics, Johns Hopkins University School of Medicine, 600 North Wolfe Street, Baltimore, MD 21287 USA; 2 Department of Chemistry and Biochemistry, University of Notre Dame, Notre Dame, IN USA; 3 Division of Gastroenterology, Valley Children’s Healthcare, Madera, CA USA; 4 USDA Children’s Nutrition Research Center, Baylor College of Medicine, and Texas Children’s Hospital, Houston, TX USA

**Keywords:** Disaccharidases deficiency, Lactase-Phlorizin hydrolase, Sucrase-Isomaltase complex, Maltase-Glucoamylase, Abdominal pain

## Abstract

**Background:**

Disaccharides such as lactose and sucrose are sugars commonly found in human diet. They are broken down by mucosal disaccharidases in the duodenum. Previous small studies found no associations between gastrointestinal (GI) symptoms and combined low disaccharidase activity. We aim to explore the associations of low activity of disaccharidase and combinations of low activity of different disaccharidases with general GI symptom presentations in a large cohort of pediatric patients.

**Methods:**

We examined a cohort (0–21 yrs.) who have undergone esophagogastroduodenoscopy and received disaccharidase activity assay from duodenal biopsy in the time period 2010 to 2012. Disaccharidase assays tested for activity of lactase, sucrase, maltase, and palatinase. GI symptoms were grouped into four categories, abdominal pain, diarrhea, weight loss, and gastroesophageal reflux.

**Results:**

Of the 347 subjects, we found an association between low lactase activity and abdominal pain (OR = 1.78; 95% CI = 1.07–2.97; *p* < 0.05). Subjects with a lactase/sucrase ratio < 0.2 were found to be associated with abdominal pain (OR = 2.25; 95% CI = 1.25–4.04; *p* < 0.05), Subjects with low pandisaccharidase may be correlated with abdominal pain and have a unique frequency of GI symptoms due to low frequency of diarrhea and weight loss, but they were not statistically significant.

**Conclusions:**

Low activities of certain disaccharidase combinations may be associated with GI symptoms in subjects; a prospective study may be needed to investigate further.

## Background

Disaccharides including lactose, maltose, sucrose, and isomaltose are a major part of the human diet, and for the past few decades the consumption of sugars has increased globally especially in children, and though decreasing in a few countries, it still remains high overall [1–4]. This trend suggests that more children may experience symptoms/medical conditions related to abnormal disaccharidase activities in the future, so the need to understand the effects of abnormal disaccharidase activities could be increasing. The enzymes studied are referred to as lactase, maltase, sucrase, and palatinase by their corresponding digestion of lactose, maltose, sucrose, and isomaltose respectively in the laboratory. Although in reality, these enzymes exist as enzyme complexes: lactase-phlorizin hydrolase (EC 3.2.1.108) hydrolyzes lactose, sucrase-isomaltase (EC 3.2.1.48) hydrolyzes sucrose and isomaltose, and maltase-glucoamylase (EC 3.2.1.20) hydrolyzes maltose and starch [5]. The isomaltase (palatinase) subunit of the sucrase-isomaltase complex hydrolyzes isomaltose while the sucrase subunit hydrolyzes sucrose. Lactase is a beta-glucosidase and maltase, sucrase, and palatinase are all alpha-glucosidases [6, 7].

Because disaccharidases are brush border enzymes predominantly present throughout the small intestine, direct measurements of disaccharidase activities are performed by small intestinal biopsy. In patients who undergo esophagogastroduodenoscopy (EGD) with biopsy, disaccharidase assays can be done along with histology analysis for comprehensive evaluation as causes of clinical gastrointestinal (GI) symptoms, such as abdominal pain, are often multifactorial so multiple tests tend to be combined in the hopes of revealing the underlining etiology for the GI symptoms.

Lactase deficiency is a common and well-studied condition. The global prevalence of lactose intolerance is estimated to be 68% (95% CI = 64–72) [8]. Lactose intolerance is known to be more prevalent in non-White races/ethnicities such as Blacks and Asians and to increase with age [8–11]. Depending on the degrees of the deficiency, the condition may result in symptoms of abdominal pain, diarrhea, nausea, flatulence, and/or bloating after consumption of lactose [12]. The epidemiology of sucrase, maltase, and palatinase deficiency is less well-known partially due to them being relatively uncommon [13, 14]. Low activity of disaccharidases has been reported in pediatric patients with persistent GI symptoms with normal small intestinal histology [15, 16]. However, a recent study by Chumpitazi et al. on low disaccharidase activity in pediatric patients with functional dyspepsia was unable to find any significant association between specific GI symptoms and low disaccharidase activity beyond low lactase [17]. We believed that the study may have had too small of a sample size to a find statistical significance. Thus, the objective of our study was to explore the association of low activity of disaccharidase and combinations of low disaccharidases with general GI symptom presentations in a larger sample size of children and young adults.

## Methods

This study was approved by the institutional review board of the Johns Hopkins School of Medicine. We performed a retrospective review of demographic and clinical data in children and young adults. The following are the inclusion/exclusion factors. Subjects less than or equal to 21 years of age who have undergone EGD with disaccharidase assays were included. All sexes, race/ethnicities, and socioeconomic backgrounds were included. Subjects who were not tested for disaccharidase activity in the time period May 2010 to March 2012 were excluded. Subjects who were not tested for disaccharidase activity from duodenal biopsy were excluded. Subjects with any villous atrophy were excluded. Subjects who did not present or undergo EGD for at least one of the four groupings of GI symptoms, abdominal pain, diarrhea, weight loss, or gastroesophageal reflux, were excluded from the study. Only one subject in our original sample population did not present with any of the four symptoms.

The clinical indications for EGD and disaccharidase assays were categorized in four GI presentations: abdominal pain (persistent/chronic), gastroesophageal reflux (included acid reflux, vomiting, regurgitation, and dysphagia), diarrhea (persistent/chronic) and weight loss (included failure to thrive and poor weight gain). Similar symptom categories have been used in related previous studies [18]. As a standard procedure, two duodenal biopsy tissues from the second portion of the duodenum were obtained during the EGD. The biopsy tissues were frozen immediately in dry ice and one of the two pieces was submitted for assays of disaccharidases (lactase, sucrase, maltase, and palatinase) and the other piece was submitted for histology analysis. All patients also had esophageal biopsies which were submitted for histology analysis. Disaccharidase assays were performed by Quest Diagnostics™, Nichols Institute, 27,027 Tourney Road, Valencia, CA 91355–5386, and reported in μM/min/g protein. The low activity of disaccharidases was defined using the reference values provided by Quest Diagnostics™ which are 15, 25, 100 and 5 μM/min/g protein for lactase, sucrase, maltase, and palatinase respectively [19]. Table 3 used categorical classifications, low versus normal enzyme activity. Lactase/sucrase ratio was also evaluated with a cutoff of 0.2 which has been reported to suggest adult type hypolactasia [20].

The remaining mucosal biopsy was submitted for histologic evaluation. Multiple histology slides were examined and reported by the Pathology Service. Biopsies were fixed in 10% neutral buffered formalin (pH 7.3) and embedded in paraffin wax, and sections were stained with hematoxylin and eosin. Crypt and villus length were estimated from well-oriented, complete, crypt-villus units. The average ratio of crypt to villus was evaluated for each biopsy. The number of theliolymphocytes was also determined along oriented villi and estimated as number of theliolymphocytes per 100 epithelial cells (% theliolymphocytes). The report of normal histology excluded all villus atrophy, defined as villus-crypt ratio less than 4, or increased theliolymphocytes greater than 20%. A separate investigation of clinically similar subjects with normal villous height but increased theliolymphocytes is in preparation. Presence of abnormal histology was also noted, such as neutrophil infiltrate, cryptitis, and mixed cell infiltrate in lamina propria, and were excluded from these series. Other histological findings such as reactive epithelial changes in distal esophagus, elevated intraepithelial lymphocytes in duodenum, eosinophilic infiltrate in duodenum, active duodenitis, and chronic duodenitis were described in the Results.

Shapiro-Wilk test showed that disaccharidase activity for all four disaccharidases did not have a normal distribution and was right-skewed, so the non-parametric Mann-Whitney *U* test was used instead of Student’s t-test for significance calculations in Table 2. After an initial overall analysis, the subjects were stratified into age groups, pre-teenager (0–11 years) and teenager (12–21 years) in Table 2. Logistic regression was used for significance calculations for Table 3 with results reported in odds ratio (OR). A multivariate model was used in order to control for sex and race of the subjects. Tests for both Tables 2 and 3 were two-tail tests. Low disaccharidase activities were described in median and frequency of GI symptoms were described as percentages. In Table 2, statistical analysis was done to test the significance of the difference in disaccharidase levels in the absence versus presence of a GI symptom. In Table 3, statistical analysis was done to test the significance of the difference in GI symptom frequency in low disaccharidase activity groups versus in GI symptom frequency in normal disaccharidase activity group. A *p*-value below 0.05 was considered statistically significant. Bonferroni correction was used to account for multiple comparisons done in Table 2. All calculations including statistical significance and confidence intervals were performed using STATA® software (StataCorp. 2013. Stata Statistical Software: Release 13. College Station, TX: StataCorp LP.). All data on the subjects were extracted from the Johns Hopkins Hospital’s patient medial record database (EPIC Clarity database).

## Results

This study included 347 subjects. 163 (47.0%) of the subjects were male and 184 (53.0%) were female. 163 (47.0%) were teenagers (12–21 years) and 184 (53.0%) were pre-teenagers (0–11 years). Self-reported race was classified as either Caucasian or non-Caucasian. 234 (67.4%) of the subjects were Caucasian and 113 (32.6%) were non-Caucasian. 223 (64.3%) of the subjects reported symptoms of abdominal pain, 119 (34.3%) gastroesophageal reflux, 97 (28.0%) diarrhea, and 39 (11.2%) weight loss (Table 1).

**Table 1:** Demographic data and clinical profiles of subjects in our study

	Characteristics	Total Number (%)
**Total**		**347 (100)**^**1**^
**Sex**	Male	164 (47.0)
	Female	184 (53.0)
**Race**	Caucasian	234 (67.4)
	Non-Caucasian	113 (32.6)
**Age**	Teenager (12–21 yrs.)	163 (47.0)
	Pre-Teenager (0–11 yrs.)	184 (53.0)
**GI Symptoms**^**2**^	Abdominal Pain	223 (64.3)
	Reflux	119 (34.3)
	Diarrhea	97 (28.0)
	Weight Loss	39 (11.2)

^1^Values in parenthesis represents percentage values

^2^The subjects are not mutually exclusive in GI symptoms

When analyzing GI symptoms as the independent variable and disaccharidase activity as the dependent variable, pediatric patients with abdominal pain was associated with lower disaccharidase activity levels for lactase and palatinase compared to the levels in subjects without abdominal pain. Abdominal pain was significantly associated with lower lactase activity (*p* < 0.001). Lower palatinase activity (*p* < 0.05) appeared to be associated with abdominal pain, but after Bonferroni correction, the result was found to be insignificant. Statistical analysis of the other GI symptoms found the differences in disaccharidase activity for subjects with the GI symptom present versus those with GI symptom absent were insignificant (*p* > 0.05) (Table 2). However, when the subjects were stratified by age group, the pre-teenage group was found to have different patterns of disaccharidase activity compared to the teenage group. In the pre-teenage group, those with abdominal pain were found to have significantly lower lactase, maltase, sucrase, and palatinase activity (*p* < 0.05) and those with weight loss had significantly higher lactase activity (*p* < 0.05) before Bonferroni correction. The teenage group had no significant differences in disaccharidase activities for subjects with the GI symptom present versus those with GI symptom absent. With Bonferroni correction, however, the differences in the pre-teenage group were all insignificant (Table 2B and Table 2C).

**Table 2:**
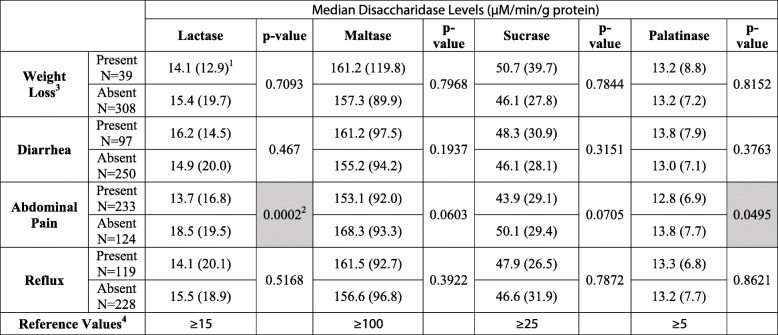
Median disaccharidase values associated with GI symptoms in all pediatric patients

^1^The values in parenthesis display interquartile range for median

^2^Shaded boxes have *p* < 0.05

^3^Patients with multiple GI symptoms were included in more than one symptom category per specific disaccharidase

^4^Reference values were added to this table only to put the results into context and were not used in statistical calculations

When analyzing low disaccharidase activity as the independent variable, sex and race as controls, and GI symptoms as the dependent variable, subjects with low enzyme activity for any disaccharidases was associated with higher frequency of abdominal pain compared to subjects with normal (≥reference values) enzyme activity for all disaccharidases (OR = 1.84; 95% CI = 1.24–2.75; *p* < 0.05). Additionally, subjects with only low lactase activity were associated with having higher frequency of abdominal pain compared to subjects with normal enzyme activity for all disaccharidases (OR = 1.76; 95% CI = 1.03–3.01; *p* < 0.05) (Table 3). Subjects with a lactase/sucrase ratio of less than 0.2 were found to be associated with the presence of abdominal pain (OR = 2.25; 95% CI = 1.25–4.04; *p* < 0.05) but it was also associated with the absence of weight loss (OR = 0.25; 95% CI = 0.07–0.83; *p* < 0.05).

**Table 3:** Frequency of GI symptoms in pediatric patients with low disaccharidase activity

Low Activity Groups	Total		Presenting Symptom Frequencies^**1**^							
			Abdominal Pain		Diarrhea		Weight Loss		Reflux	
	N	%	N	%	N	%	N	%	N	%
Total Population	347	100.0	223	64.3	97	28.0	39	11.2	119	34.3
Total Normal Activity	174	50.1	101	58.4	56	32.4	23	13.3	59	34.1
Any Low Activity^2^	173	49.9	122	70.5	40	23.1	16	9.2	60	34.7
**Low Beta-Glucosidase alone**										
**Lactase Alone**	111	32.0	79	71.2	27	24.3	7	6.3	39	35.1
**Low Beta-Glucosidase + Low Alpha-Glucosidases Combined**										
Sucrase+Lactase	5	1.4	4	80.0	2	40.0	1	20.0	1	20.0
Maltase+Lactase	16	4.6	11	68.8	5	31.3	1	6.3	7	43.8
Sucrase+Lactase+Maltase	24	6.9	16	66.7	6	25.0	5	20.8	10	41.7
Pandissacharidase^3^	8	2.3	7	87.5	1	12.5	0	0.0	1	12.5
**Total**	53	15.3	38	71.7	14	26.4	7	13.2	19	35.8
**Low Alpha-Glucosidases alone**										
Maltase alone	3	0.9	1	33.3	0	0.0	1	33.3	1	33.3
Maltase+Sucrase	4	1.2	4	100.0	0	0.0	0	0.0	0	0.0
Sucrase+Maltase+Palatinase	2	0.6	0	0.0	0	0.0	2	100.0	0	0.0
**Total**	9	2.6	5	55.6	0	0.0	3	33.3	1	11.1

^1^Percentages in the “Presenting Symptom Frequencies” columns are percentages of N of the “Total” column. Patients with multiple GI symptoms were included in more than one symptom category per specific disaccharidase

^2^Reference values are 15, 25, 100 and 5 μM/min/g protein for lactase, sucrase, maltase and palatinase respectively

^3^Pandisaccharidase = Maltase+Sucrase+Lactase+Palatinase

Subjects with low pandisaccharidase (all four tested enzymes) activity may be correlated with increased frequency of abdominal pain compared to subjects with normal enzyme activity for all disaccharidases, but this difference was not statistically significant (OR = 4.87; 95% CI = 0.59–40.58; *p* = 0.14). Other disaccharidases alone or combined disaccharidases with low activity did not show statistical significance for changes in GI symptom frequency compared to normal disaccharidase activity (Table 3).

Of the 347 subjects, 209 (60%) had reactive epithelial changes in the distal esophagus, 34 (10%) chronic duodenitis, 19 (5%) had elevated intraepithelial lymphocytes in duodenum, 7 (2%) had active duodenitis 5 (1%) had eosinophilic infiltrate in the duodenum, and 0 (0%) had villous atrophy or attenuation of villous height. Having low lactase activity with at least one low alpha-glucosidase activity was associated with increased odds of having chronic duodenitis (OR = 3.40; 95% CI = 1.34–8.62; *p* < 0.05). There were no other significant associations between the histology and having low enzyme activity in any of the four disaccharidases.

## Discussion

This retrospective study on disaccharidase activity in children and young adults suggests that certain GI symptoms could be associated with low disaccharidase activity. We found associations between low lactase activity and abdominal pain, and vice-versa. Abdominal pain was also found to be associated with lower palatinase activity. Although not statistically significant, low pandisaccharidase activity may be associated with a unique pattern of GI symptoms, higher frequency of abdominal pain and lower frequency of diarrhea, gastroesophageal reflux, and weight loss.

We found the association of low lactase activity alone in subjects with higher frequency of abdominal pain when compared to subjects with all normal disaccharidase activity, to be statistically significant (Table 3). This finding is similar to reports by others stating that in older children and teenagers, lactose intolerance is a common cause of abdominal pain [12, 21]. However, there are important differences between our study of low lactase activity and previous studies of lactose intolerance on GI symptoms. Lactose intolerance is a clinical syndrome diagnosed by adverse reactions due to ingestion of lactose; it is not necessarily related to low lactase activity level or having low lactase activity by our standards. In addition, we found upon comparison of subjects with any low disaccharidase activity to subjects with all normal disaccharidase activity, the frequency of abdominal pain was significantly higher in subjects with any low disaccharidase activity. This result, however, may have been skewed because children with low lactase activity alone made up 64% of the subjects with any low disaccharidase activity. Our finding that low lactase activity alone was the most common in our cohort is consistent with previous reports [13, 22].

Subjects with low pandisaccharidase activity may be correlated to higher frequency of abdominal pain (87.5%), but interestingly, lower frequency of diarrhea (12.5%), gastroesophageal reflux (12.5%), and weight loss (0%) compared to subjects with all normal disaccharidase activity (Table 3). This observation appeared to be unique, however, statistical significance was not achieved, likely due to inadequate sample size. A recent study found a moderately similar pattern to ours. In a study of 21 children with pandisaccharidase deficiency, Cohen et al. found that 95% had abdominal pain, 42% had diarrhea, and 38% had poor weight gain [18]. We also believe that the discrepancy in the frequency of diarrhea and poor weight gain (included in weight loss for our study) could be due to the small sample size in our study. Additionally, we suspected that the frequencies of GI symptoms in subjects with only low lactase activity may be different from the frequencies in subjects low pandisaccharidase activity, but this also was not statistically significant.

Our findings strongly suggest that pediatric patients with abdominal pain present is associated with lower lactase activity compared to those with abdominal pain absent. This association had statistical significance (*p* < 0.001). There also appeared to be a relationship of the presence of abdominal pain with lower maltase, sucrase, and palatinase activity, but the reduction did not have statistical significance (Table 2). Because both sucrose and isomaltose are catalyzed by the same enzyme complex, sucrase-isomaltase, it may be expected that a reduction in palatinase activity would also correspond to a reduction in sucrase activity in the same person. However, sucrose and isomaltose are catalyzed at different active sites, located on different enzymes of the enzyme complex, which function independently of each other [23, 24]. This suggests that minor genetic mutations that alter the activity of one of the enzymes on the complex may not affect the other, or at least to the same degree [25]. When the cohort was stratified by age group, differences in disaccharidase activity by GI symptoms were only present in the pre-teenage group suggesting that younger patients may be more sensitive to disaccharidase activity abnormalities or have not had time to adapt to living with reduced disaccharidase activity.

In our study, we found chronic duodenitis to be associated with having both low lactase and low alpha-glucosidases. Although it is known that cow’s milk allergy can cause duodenitis, lactase deficiency has not been reported to be associated with duodenitis [26]. Wiecek et al. found decreased lactase activity in children with inflammatory bowel disease, however, they did not examine children without inflammatory bowel disease to see if the prevalence was different [27]. In our study, we did not find any association with lactase alone, but with lactase in combination with other disaccharidase deficiencies which has not been examined before in relation to chronic duodenitis. This may suggest that chronic duodenitis may give different degrees of inflammation of entire the villous structure, affecting not only lactase on the tip, but alpha-glucosidases in the central structure of the villi. Because our study focuses mainly on the association of low disaccharidase activity to clinical symptoms in pediatric patients who had no villous atrophy, we do not claim that low disaccharidase activity is the cause of the GI symptoms experienced in our subjects.

In our study, there were a few limitations to consider. First, low activity of an enzyme is not equal to a clinical enzyme deficiency. Second, not all subjects had additional tests, such as hydrogen breath tests using lactose as a substrate, to confirm the reduction of a disaccharidase. Third, this was a cross-sectional study so there may have been some external factors, which affected a one-time result of activity of one disaccharidase or more, for example, transportation time which may artificially lower disaccharidase activity due to enzyme decomposition over time. However, because we made comparisons within our large group of subjects whose biopsy samples likely faced similar conditions, we believe that the effects of these mentioned limitations on our findings to be minimal. Since this is a retrospective study, we do not have clinical outcome data on restriction of corresponding disaccharides or polysaccharides such as lactose, sucrose, or starch. However, despite having been started 8 years ago, this study would be the largest of its kind [17].

## Conclusions

In summary, we found that low lactase activity is closely associated with abdominal pain and vice versa for relative lactase activity. Additionally, abdominal pain is associated with lower palatinase activity, but not vice versa for low palatinase activity as with low lactase activity. Subjects with a lactase/sucrase ratio less than 0.2 were found to be associated with abdominal pain suggesting adult-type hypolactasia, and subjects with both low lactase and low alpha-glucosidase were found to be associated with having chronic duodenitis. Low pandisaccharidase activity also appears to be associated with high frequency of abdominal pain, but it was not statistically significant. However, further studies with larger sample sizes should be done to reveal more associations and determine whether these associations may be useful in diagnosis.

## Data Availability

The datasets generated during and/or analyzed during the current study are not publicly available due to confidentiality of human subjects but are available from the corresponding author on reasonable request.

## Abbreviations

CI: Confidence interval
EGD: Esophagogastroduodenoscopy
GI: Gastrointestinal
OR: Odds ratio
